# Oxaliplatin-DNA adduct formation in white blood cells of cancer patients

**DOI:** 10.1038/sj.bjc.6604387

**Published:** 2008-05-27

**Authors:** A C Pieck, A Drescher, K G Wiesmann, J Messerschmidt, G Weber, D Strumberg, R A Hilger, M E Scheulen, U Jaehde, in collaboration with Central European Society for Anticancer Drug Research-EWIV (CESAR)

**Affiliations:** 1 Institute of Pharmacy, Department of Clinical Pharmacy, University of Bonn, Bonn, Germany; 2 ISAS–Institute for Analytical Sciences, Department of Metabolomics, Dortmund, Germany; 3 Department of Internal Medicine (Cancer Research), West German Cancer Centre, University of Essen, Essen, Germany

**Keywords:** DNA adducts, neurotoxicity, oxaliplatin, pharmacokinetics, pharmacodynamics, platinum

## Abstract

In this study, we investigated the kinetics of oxaliplatin-DNA adduct formation in white blood cells of cancer patients in relation to efficacy as well as oxaliplatin-associated neurotoxicity. Thirty-seven patients with various solid tumours received 130 mg m^−2^ oxaliplatin as a 2-h infusion. Oxaliplatin-DNA adduct levels were measured in the first cycle using adsorptive stripping voltammetry. Platinum concentrations were measured in ultrafiltrate and plasma using a validated flameless atomic absorption spectrometry method. DNA adduct levels showed a characteristic time course, but were not correlated to platinum pharmacokinetics and varied considerably among individuals. In patients showing tumour response, adduct levels after 24 and 48 h were significantly higher than in nonresponders. Oxaliplatin-induced neurotoxicity was more pronounced but was not significantly different in patients with high adduct levels. The potential of oxaliplatin-DNA adduct measurements as pharmacodynamic end point should be further investigated in future trials.

Oxaliplatin is a third-generation diaminocyclohexane (DACH)-platinum complex with lacking cross-resistance against cisplatin and carboplatin. Oxaliplatin is part of the therapeutic standard regimens for the treatment of metastatic colorectal cancer in combination with fluorouracil/leucovorin (Giacchetti *et al*, 2000; de Gramont *et al*, 2000). Oxaliplatin has also been used as a single agent in this disease and other malignancies (Faivre *et al*, 1999; Germann *et al*, 1999; Garufi *et al*, 2001). Besides treatment of patients with metastatic disease, oxaliplatin is also approved for adjuvant protocols (André *et al*, 2004). However, neurotoxicity in the form of transient neuropathy and a persistent cumulative typical sensory polyneuropathy is common and dose-limiting, whereas other types of toxicity associated with platinum complexes are rare or of minor severity (Cassidy and Misset, 2002; Gamelin *et al*, 2002; Grothey, 2003).

Similar to other platinum coordination complexes, the cytotoxic activity is based upon the formation of mono- or bifunctional adducts with DNA (Scheeff *et al*, 1999; Lévi *et al*, 2000). Among other factors, the platinum-DNA adduct levels are influenced by drug uptake, drug efflux and DNA repair (Fink *et al*, 1997; Raymond *et al*, 2002; Faivre *et al*, 2003). As the degree of DNA platination plays a central role in the mechanism of action of platinum complexes, the determination of platinum-DNA adducts formed in the tumour tissue might be of interest for individual dose adaptation. However, the poor accessibility of tumour tissue is a major obstacle for routine measurement. Therefore, white blood cells (WBC) have been considered as surrogate cells (Reed *et al*, 1988; Poirier *et al*, 1992; Nadin *et al*, 2006). Various clinical studies with cisplatin have shown that tumour response is related to platinum-DNA adduct levels in WBC (Reed *et al*, 1987; Parker *et al*, 1991; Schellens *et al*, 1996).

The platinum-DNA adduct formation in WBC was found to be highly predictive for tumour response after platinum-based therapy and even more predictive than platinum pretreatment, stage of disease, histological type and tumour grading (Reed *et al*, 1990). Recently, a significantly better disease-free survival was reported for cisplatin-treated head and neck carcinoma patients with higher adduct levels (Hoebers *et al*, 2006). The feasibility of intraindividual dose escalation of cisplatin based on platinum-DNA adduct levels has been shown in two clinical trials in patients with head and neck cancer (Schellens *et al*, 2001) and non-small cell lung cancer (Schellens *et al*, 2003). However, not all investigators found a relationship between adduct levels and tumour response. It has been speculated that the technique of the adduct measurement, the tumour types investigated, and medication factors could have influenced the adduct levels and led to conflicting results (Motzer *et al*, 1994; Bonetti *et al*, 1996).

For oxaliplatin, only two reports were published on adduct levels, each in six patients (Allain *et al*, 1996; Liu *et al*, 2002). Studies investigating a larger number of patients and a potential relationship to tumour response and toxicity have not been performed as yet. For the first time, we report here the platinum-DNA adduct levels in WBC of patients receiving oxaliplatin in the context of a clinical study.

Measurement of platinum-DNA adduct levels in WBC has been successfully applied in cancer patients receiving cisplatin and high-dose carboplatin using flameless atomic absorption spectrometry (Kloft *et al*, 1999b). Because of the generally lower extent of platinum-DNA adduct formation after administration of oxaliplatin (Raymond *et al*, 2002), we used the more sensitive adsorptive stripping voltammetry to quantify DNA-bound platinum (Weber *et al*, 2004).

This study was designed to assess both time-dependence and interindividual variability of platinum-DNA adduct formation in relation to pharmacokinetics in plasma and ultrafiltrate after administration of oxaliplatin as well as to detect potential relationships with efficacy and/or toxicity.

## PATIENTS AND METHODS

### Patients

Blood sampling was performed within a single-centre, open-label, non-placebo-controlled, nonrandomised phase I study that was conducted to investigate the safety, pharmacokinetics and efficacy of sorafenib (BAY 43-9006), a multikinase inhibitor, in combination with oxaliplatin (Kupsch *et al*, 2005). The study protocol was approved by the local ethical committee. All procedures were in accordance with the Helsinki Declaration of 1975 (as revised in 2000).

The patients included in this study exhibited advanced refractory solid tumours for which no standard therapy existed and for whom treatment with oxaliplatin was considered acceptable. Other eligibility criteria were age >18 years, a life expectancy of at least 12 weeks, and an adequate bone marrow, liver and renal function. Renal function was characterised by creatinine clearance estimated by the formula of Cockcroft and Gault. Patients should not have received oxaliplatin within 3 months before enrolment. Patient characteristics are summarised in Table 1.

### Therapy

Thirty-seven patients were treated with 130 mg m^−2^ oxaliplatin every 3 weeks combined with different doses of sorafenib. Oxaliplatin was administered as 2-h i.v. infusion. Blood samples were collected during the first treatment cycle. Sorafenib was given twice a day from the fourth day of the first cycle onwards, that is sorafenib had not been co-administered when blood samples were collected for this study.

### Assessment of response and toxicity

Response was assessed according to RECIST after the second treatment cycle, that is 6 weeks after start of treatment (Therasse *et al*, 2000). Owing to the relatively early assessment of response, a 15–29% decrease in the sum of the longest diameters of target lesions was split out of the ‘stable disease’ (SD) category and defined in deviation from the original RECIST criteria as ‘minor response’ (MR). ‘Stable disease’ was then defined as neither sufficient shrinkage to qualify for MR nor sufficient increase to qualify for ‘progressive disease’ (PD). In the following, patients exhibiting partial or minor remission were regarded as ‘responders’ and those exhibiting SD or PD were regarded as ‘nonresponders’. The treatment-associated toxicity was assessed after each treatment cycle according to the Common Toxicity Criteria (National Cancer Institute, 1999). Oxaliplatin-specific neuropathy was evaluated by the scale of Lévi *et al* (1992).

### Analysis of platinum in plasma and ultrafiltrated plasma

Before, during and after administration of oxaliplatin, 13 blood samples were collected and cooled and the plasma was separated by centrifugation (3200 **g** for 5 min at 4°C) within 30 min. For ultrafiltration, 1 ml of plasma was transferred to a Centrisart™ ultrafiltration system (Sartorius AG, Göttingen, Germany; cutoff 10.000) and centrifuged for 20 min at 2000 **g** and 4°C. All samples were immediately frozen and stored at −20°C until further analysis.

Elemental platinum in plasma and ultrafiltrate was determined by flameless atomic absorption spectrometry using a modification of a procedure described by Kloft *et al* (1999a). In brief, an atomic absorption spectrometer (SpectrAA™ Zeeman 220; Varian, Darmstadt, Germany) equipped with a graphite tube atomisator and a platinum hollow cathode lamp was used. The temperature programme was optimised for each matrix and concentration range. The method was validated and met the international requirements on bioanalytical methods (Shah *et al*, 2000; US Department of Health and Human Services, FDA, CDER and CVM, 2001; Gastl *et al*, 2003).

### Determination of platinum-DNA adducts in WBC

DNA platination in WBC was determined by a four-step procedure consisting of isolation of WBC out of whole blood, separation of DNA, quantification of DNA and quantification of platinum bound to DNA using a modification of the method by Kloft *et al* (1999b).

In brief, WBC were isolated 0, 4, 24 and 48 h after the start of oxaliplatin infusion within 2 h after blood collection using density gradient centrifugation (30 min at 400 **g** and room temperature using Polymorphprep™; Axis-Shield, Oslo, Norway). Two bands (mononuclear and polymorphonuclear cells) were harvested and pooled. Then the cells were washed twice with ice-cold PBS to remove other blood components and the gradient medium. WBC samples were immediately frozen and stored at −20°C until further analysis.

The isolation of DNA out of WBC was performed by solid-phase extraction with QIAamp™ DNA-blood kits (Qiagen, Hilden, Germany). The isolation procedure consisted of the lysis of WBC and adsorption of DNA to a silica membrane followed by two washing steps to remove other cell components. In the last step, DNA was eluted from the column. All DNA samples were stored at −20°C until further analysis. The DNA concentrations and the purity of the isolated DNA were determined by UV spectrometry measuring the absorption at 260, 280 and 320 nm. This method was validated and met the requirements on bioanalytical methods.

The quantification of platinum bound to DNA was performed by a validated adsorptive stripping voltammetry method. This highly sensitive method, described by Weber *et al* (2004), allowed the determination of platinum with a lower limit of quantification of 0.4 pg ml^−1^ (Messerschmidt *et al*, 1992; Gelevert *et al*, 2001). In brief, the residue of the dried eluate was decomposed to mineralisation using a high-pressure asher (HPA, Kürner, Rosenheim, Germany). A detailed description of the mineralisation process is given by Messerschmidt *et al* (1992). Platinum was then quantified by adsorptive stripping voltammetry using a Metrohm Polarecord 626 (Metrohm, Herisau, Switzerland). The concentration of platinum was evaluated from three standard additions with sufficient accuracy and precision (relative error <9.8% and relative standard deviation <8.0%). On the basis of the DNA and platinum concentrations, the platinum-nucleotide ratio was calculated using the relative atomic mass of platinum (A_*r*_(platinum) =195.1) and the relative molecular mass of nucleotides (M_*r*_(nucleotide) =330).

The between-day precision for the whole method consisting of DNA isolation, DNA and platinum quantification was 11.8% (relative standard deviation). On the basis of this result, the method was regarded as being suitable for characterizing platinum-DNA adduct formation and its interindividual variability in clinical samples.

### Pharmacokinetic data analysis

Individual pharmacokinetic parameters were estimated using a compartmental approach by means of the validated software WinNonlin™ 4.0. (Pharsight Corporation, Mountain View, CA, USA). The following parameters were estimated by using a two-compartment model: AUC_∞_, CL, *V*_ss_, *t*_1/2*λ*1_ and *t*_1/2z_. The peak concentration (*C*_max_) and the time until the peak concentration was reached (*t*_max_) were taken directly from the concentration-time profile.

Besides, the area under the adduct curve (AUA_0−48 h_) and the AUC_0−48 h_ were determined using the linear trapezoidal rule.

### Statistical analysis

The statistical analysis was performed using the software SPSS 12.0.1 for Windows. Data distribution was tested by the Shapiro-Wilk test. For comparisons between groups of patients, for example, responders *vs* nonresponders, the Mann–Whitney test was used. The correlation between pharmacokinetic parameters and adduct levels was assessed by the coefficient of correlation according to Kendall.

## RESULTS

### Platinum pharmacokinetics in plasma and ultrafiltrate

**Figure 1:**
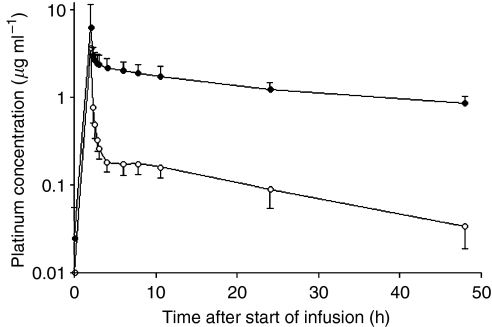
Mean platinum concentration-time profiles (mean±s.d.; *n*=37) in plasma (•) and ultrafiltrate (○).

Figure 1 shows the platinum concentration-time course during the first 48 h after the start of oxaliplatin infusion in plasma as well as ultrafiltrate during the first treatment cycle. The pharmacokinetic parameters are summarised in Table 2. In ultrafiltrate, the AUC_∞_ was about 9% compared to the AUC_∞_ in plasma.

### Time course of platinum-DNA adduct formation in WBC

**Figure 2:**
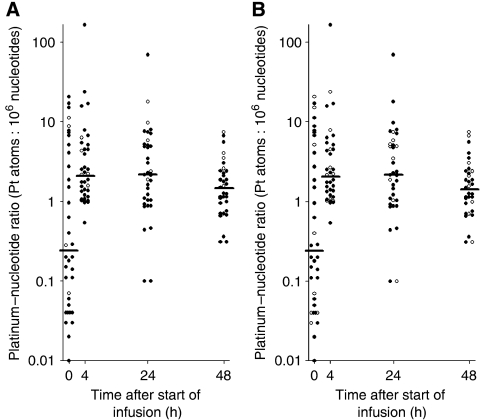
Individual and median platinum-nucleotide ratios (*n*=37); (**A**) of nonresponders (•) and responders (○); (**B**) of patients with low (0–1; •) and high (2–4; ○) grade of neurotoxicity.

Figure 2 shows the individual platinum-nucleotide ratios including the median at the different time points 0 h (A0 h), 4 h (A4 h), 24 h (A24 h) and 48 h (A48 h). Before the first oxaliplatin infusion, platinum adducts were detectable in several patients and this was attributed to platinum-containing pretreatment. The maximum platinum-nucleotide ratios (*A*_max_) were observed either 4 or 24 h after the start of infusion in most of the patients. The median *A*_max_ value was 4.43 Pt atoms: 10^6^ nucleotides. On the basis of the platinum-nucleotide ratios, the area under the adduct curve (AUA_0−48 h_) was calculated. The median AUA_0−48 h_ was found to be 163 Pt atoms·h: 10^6^ nucleotides.

The interindividual variability of platinum-nucleotide ratios at all times of observation was considerably higher than that of the plasma concentrations. The amount of platinum-DNA adducts was not affected by sex or other factors such as age, height, weight, body surface area, body mass index, creatinine clearance and, most interestingly, platinum pretreatment.

**Figure 3:**
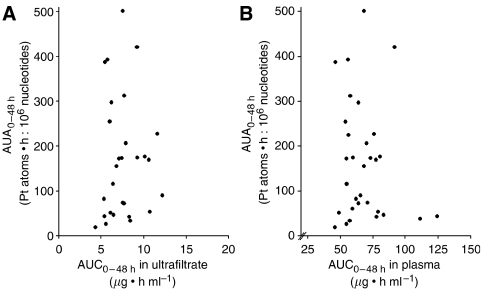
Relationship between area under the platinum-nucleotide adduct curve (AUA_0−48 h_) and AUC_0−48 h_ in ultrafiltrate (**A**) and plasma (**B**).

The relationship between pharmacokinetic parameters (AUC_0−48 h_, C_max_ and CL in plasma/ultrafiltrate) and adduct levels (A4 h, A24 h, A48 h, AUA_0−48 h_, A_max_) was examined by means of a correlation analysis. Figure 3 shows the relationship between area under the platinum-nucleotide adduct curve (AUA_0−48 h_) and AUC_0−48 h_ in ultrafiltrate (Figure 3A) and plasma (Figure 3B). The correlation coefficients according to Kendall were 0.041 and −0.065 for ultrafiltrate and plasma, respectively. Moreover, A_max_ was neither correlated to C_max_ in ultrafiltrate (*r*=−0.158) nor to C_max_ in plasma (*r*=−0.170).

### Response and toxicity

In total, 37 patients were included in this study. Thirty-one patients received a second treatment cycle and were therefore evaluable for response. Five out of 31 patients (16.1%) experienced either partial (one patient) or minor response (four patients). Of the patients, 45% experienced a stabilisation of previous PD (14 patients) and 39% showed tumour progression (12 patients).

With regard to toxicity, which was assessed after each cycle, all 37 patients were evaluable for haemato-, nephro- and hepatotoxicity in the first cycle. In general, the treatment was well tolerated. Only eight patients (21.6%) experienced toxic effects of grade 3 during the first cycle and no patient showed toxicity of grade 4.

Peripheral neurotoxicity, which is typical for oxaliplatin, was observed in most of the patients (all 37 patients were evaluable). Grade 1 neurotoxicity was observed in 75.7%, grade 2 in 16.2% and grade 3 in 8.1% of the patients. No patient experienced grade 4 toxicity.

### Relationships between adduct levels and clinical outcome

**Figure 4:**
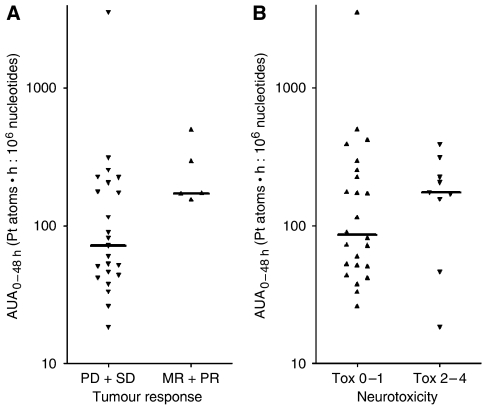
Individual and median platinum-nucleotide area under the adduct curve (AUA_0–48 h_); (**A**) of nonresponders (*n*=23) and responders (*n*=5); (**B**) of patients with low (0–1; *n*=9) and high (2–4; *n*=24) grade of neurotoxicity.

**Figure 5:**
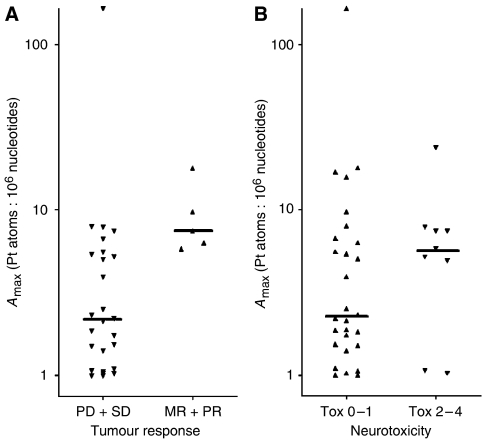
Individual and median maximum platinum-nucleotide ratio (A_max_); (**A**) of nonresponders (*n*=26) and responders (*n*=5); (**B**) of patients with low (0–1; *n*=9) and high (2–4; *n*=28) grade of neurotoxicity.

The relationships between adduct levels and response as well as the extent of neurotoxic symptoms were analysed. The results are shown in Tables 3 and 4. Platinum-nucleotide ratios at time points 24 and 48 h after the start of infusion were significantly higher in responders compared to nonresponders (for individual data points, see Figure 2A). Forty-eight hours after the start of infusion, responders reached a median platinum-nucleotide ratio that was threefold higher than in nonresponders (*P*=0.007). Besides, A_max_ was significantly different between patients with and without response (*P*=0.006). AUA_0−48 h_ was higher in responders, but the difference did not reach statistical significance. Figure 4 shows that all patients with AUA_0−48 h_ values lower than 140 Pt atoms·h: 10^6^ nucleotides (Figure 4A), as well as patients with A_max_ values lower than 4 Pt atoms: 10^6^ nucleotides (Figure 5A), were nonresponders.

In patients with grade 2–4 neurotoxicity, median adduct levels A_max_ and AUA_0−48 h_ were higher compared to patients who experienced no or mild neurotoxicity (Table 4), which is also obvious from the respective graphical presentation of individual data (Figure 2B). However, the difference did not reach statistical significance in any of the adduct parameters (Figure 4B and 5B).

## DISCUSSION

This study was conducted to characterise the kinetics of platinum-DNA adduct formation in WBC after administration of oxaliplatin and to explore the relation between adduct formation and clinical effects. Although there are numerous clinical studies on the platinum-DNA adduct formation after cisplatin and carboplatin there are only preliminary data available on oxaliplatin in a few patients (Allain *et al*, 1996; Liu *et al*, 2002).

As oxaliplatin forms considerably less adducts after therapeutic doses than the other two platinum complexes, an assay with higher sensitivity was required. Therefore, we chose adsorptive voltammetry, which allows the quantification of platinum concentrations down to 0.4 pg ml^−1^, that is, 0.05 platinum atoms: 10^6^ nucleotides can be measured in a sample of 70 *μ*g DNA (Weber *et al*, 2004). DNA quantification as well as platinum measurements in DNA samples were validated and showed sufficient accuracy and precision.

In the two published investigations dealing with the platinum-DNA adduct formation after administration of oxaliplatin, the ICP-MS technique was used for the determination of DNA-bound platinum (Allain *et al*, 1996; Liu *et al*, 2002). The platinum-nucleotide ratios measured in our study were in the same range of the data presented by Allain *et al* (1996) who examined six patients in two cycles. In the study of Liu *et al* (2002), however, the platinum-nucleotide values were 1000 times higher than in our investigation and the study of Allain *et al* (1996), although only 60 mg m^−2^ oxaliplatin were administered. According to the authors, the considerably higher adduct values were caused by the fact that not only DNA adducts but also protein adducts were measured. Therefore, the data of Liu *et al* (2002) are probably artefacts and cannot be compared with our study.

Various parameters can be derived from platinum-DNA adduct levels. Besides the maximum adduct level (A_max_), the area under the adduct curve (AUA) was calculated to characterise the DNA platination over the whole period of observation. This parameter was used by Schellens *et al* (1996) and Veal *et al* (2001) after cisplatin administration. Other authors used the adduct values measured at a certain time (Dabholkar *et al*, 1992; Gupta-Burt *et al*, 1993; Boffetta *et al*, 1998), the maximum of all values measured in several cycles (Reed *et al*, 1988) or the measurability of adducts (Reed *et al*, 1986; Poirier *et al*, 1987). The interindividual variability of adduct levels after administration of oxaliplatin was large, especially in comparison to the pharmacokinetic parameters. Large interindividual differences were also observed after administration of cisplatin and carboplatin (Reed *et al*, 1993). As sorafenib was co-administered only from day 4 of the first cycle onwards, the effect of sorafenib on adduct formation can be excluded in our study.

So far, patient-individual factors influencing the extent of DNA platination were investigated only in a small number of patients for cisplatin and carboplatin. One possible parameter that could influence adduct formation is platinum exposure in ultrafiltrate. The results of previous studies investigating a possible correlation between pharmacokinetic parameters in ultrafiltrate and adduct parameters were not consistent. Peng *et al* (1997) and Veal *et al* (2001) did not observe any correlation between platinum pharmacokinetics and adduct formation except for cisplatin at one particular time point. In contrast, Schellens *et al* (1996) found a strong correlation between AUC_UF_ and AUA (*r*=0.78; *P*<0.0001). Recently, a correlation between carboplatin AUC and platinum-DNA adduct levels was reported after high-dose carboplatin in children (Veal *et al*, 2007). These contradictory results indicate the importance of intracellular processes, for example, cellular uptake, inactivation by glutathione and DNA repair. Another factor that may have influenced the adduct levels is platinum pretreatment, particularly as some patients in our study exhibited measurable adduct levels before oxaliplatin administration. However, pretreated patients did not show higher adduct parameters (measured adduct levels at any time, A_max_ and AUA) than those patients who were not pretreated with platinum complexes. In addition, demographic factors were examined concerning their influence on adduct formation, for example, sex and age. However, no correlation was found for any of these patient characteristics (Fichtinger-Schepman *et al*, 1990; Veal *et al*, 2001).

There are various reports on the possible relationships between DNA platination and tumour response after cisplatin- and carboplatin-based chemotherapy. In most of the studies, a large variability of adduct levels was observed, leading to overlapping ranges of adduct values for responders and nonresponders. Nevertheless, in some studies, significant differences concerning the extent of DNA platination between both groups were shown (Reed *et al*, 1987, 1988, 1993; Parker *et al*, 1991; Schellens *et al*, 1996). In contrast, other authors did not find a positive correlation between adduct formation and response (Gupta-Burt *et al*, 1993; Motzer *et al*, 1994; Boffetta *et al*, 1998). In our study, the adduct levels 24 and 48 h as well as A_max_ were correlated to response. A_4 h_ and AUA_0−48 h_ were considerably higher in responders than in patients with stable or progressive disease, but statistical significance was not reached. It is remarkable that all patients with AUA_0−48 h_ values lower than 140 Pt atoms·h: 10^6^ nucleotides were nonresponders. Provided that a dose-dependence of oxaliplatin-DNA adduct formation can be shown, an AUA in this range may serve as a target value for individual dose escalation to increase the probability of a tumour response. However, this has to be confirmed in a larger group of patients. Moreover, adduct levels should be investigated in a more homogeneous patient population, especially with regard to the tumour entity and stage.

With regard to a possible correlation to toxicity, we focused on the neurotoxicity of oxaliplatin, which is often dose-limiting. In our study, only the acute peripheral neurotoxicity was observed because a cumulative dose of 700 mg m^−2^, after which the chronic sensory neurotoxicity normally occurs, was not reached. Although patients with weak or no neurotoxic symptoms exhibited smaller adduct values than patients with a peripheral neurotoxicity grade 2–4, the difference was not significant. One may speculate that in a larger group of patients, significance would have been found. Nevertheless, the association between adduct levels and neurotoxicity seems to be weaker compared with the relationship between adduct levels and tumour response. The results of studies investigating the correlation between DNA platination and toxicity after cisplatin- or carboplatin-based treatment were inconsistent. In two studies no correlation was found (Bonetti *et al*, 1996; Ghazal-Aswad *et al*, 1999), whereas others reported an association between DNA platination and haematotoxicity. High platinum-adduct levels were associated with a high degree of thrombocytopaenia (Schellens *et al*, 1996) and leukopaenia (Veal *et al*, 2001). However, the administration schemes, the tumour entities of the patients and the analytical methods used were different, which may explain these conflicting results.

In conclusion, relationships between oxaliplatin-DNA adduct formation and clinical efficacy/toxicity were analysed for the first time. The observed correlation between adduct levels and response indicates the potential of these measurements to serve as pharmacodynamic end point in clinical trials. It seems to be worthwhile to study platinum-DNA adduct formation in a larger group of patients to define target adduct values that may be used for individual dose escalation.

## Figures and Tables

**Table 1:** Patient characteristics

**Characteristic**	**No. of patients**
*Total number of patients*	37
	
*Sex*	
Male	26
Female	11
	
*Age (years)*	
Median	59
Range	32–80
	
*Height (cm)*	
Median	170
Range	159–190
	
*Mass (kg)*	
Median	73
Range	55–106
	
*Body surface area (m^2^)*	
Median	1.9
Range	1.5–2.2
	
*Body mass index (kg m* ^−^ *^2^)*	
Median	25
Range	19–34
	
*Creatinine clearance (ml min*^−*1*^)	
Median	96
Range	57–239
	
*Number of patients with pretreatment chemotherapy*	
Containing platinum complexes	28
Containing oxaliplatin	19
	
*Number of pretreatment chemotherapy regimens*	
Median	3
Range	0–5
	
*Disease site*	
Colorectal carcinoma	18
Uvea melanoma	5
Gastric cancer	4
Cancer of unknown primary origin	3
Various malignancies	7

**Table 2:** Platinum pharmacokinetic parameters during the first cycle (Mean±s.d.; *n*=37)

	***C*_max_ (*μ*g ml^−1^)**	**AUC_∞_ (*μ*g·h ml^−1^)**	***t*_1/2*λ*1_ (h)**	***t*_1/2*z*_ (h)**	**CL (L h^−1^)**	***V*_ss_ (L)**
Ultrafiltrate	6.69±12.5	9.45±5.11	0.22±0.08	18.7±5.2	14.8±5.02	255±92
Plasma	9.32±13.5	108±25	0.45±0.46	33.7±6.7	1.19±0.40	54.9±18.7

**Table 3:** Comparison of adduct parameters of nonresponders (*n*=26) and responders (*n* =5) (Medians with minimum and maximum values)

	**Nonresponders (*n*=26a,^b^)**	**Responders (*n*=5)**	***P*-value^c^**
A4 h (Pt atoms : 10^6^ nucl.)	1.48 (0.54–165)	2.23 (1.57–6.34)	0.280
A24 h (Pt atoms : 10^6^ nucl.)^a^	1.62 (0.10–69.4)	5.83 (1.88–18.0)	0.037^*^
A48 h (Pt atoms : 10^6^ nucl.)^b^	1.14 (0.31–6.69)	3.51 (1.56–7.46)	0.007^*^
A_max_ (Pt atoms : 10^6^ nucl.)	2.18 (1.00–165)	7.46 (5.83–18.0)	0.006^*^
AUA_c–48 h_ (Pt atoms·h:10^6^ nucl.)^b^	72.0 (18.5–3545)	173 (156–501)	0.071

^*^Marks a significant result (*P*<0.05).

a The A_24 h_ sample of one nonresponder was not collected.

b The A_48 h_ samples of three nonresponders were not collected.

c Mann–Whitney *U*-test.

**Table 4:** Comparison of adduct parameters of patients with low (*n*=28) or high grade neurotoxicity (*n*=9) (Medians with minimum and maximum values)

	**Grade 0–1 (*n*=28a,^b^)**	**Grade 2–4 (*n*=9)**	***P*-value^c^**
A4 h (Pt atoms : 10^6^ nucl.)	1.81 (0.54–165)	2.23 (1.02–23.7)	0.876
A24 h (Pt atoms : 10^6^ nucl.)^a^	2.14 (0.10–69.4)	4.91 (0.10–7.47)	0.428
A48 h (Pt atoms : 10^6^ nucl.)^b^	1.25 (0.31–5.60)	2.11 (0.31–7.46)	0.370
A_max_ (Pt atoms : 10^6^ nucl.)	2.27 (1.00–165)	5.83 (1.03–23.7)	0.319
AUA_0-48 h_ (Pt atoms·h: 10^6^ nucl.)^b^	86.0 (26.2–3545)	173 (18.5–387)	0.592

a The A_24 h_-sample of one patient with low grade neurotoxicity was not collected.

b The A_48 h_-samples of four patients with low grade neurotoxicity were not collected.

c Mann–Whitney *U*-test.
